# Genome Editing Technology for Genetic Amelioration of Fruits and Vegetables for Alleviating Post-Harvest Loss

**DOI:** 10.3390/bioengineering9040176

**Published:** 2022-04-18

**Authors:** Chanchal Kumari, Megha Sharma, Vinay Kumar, Rajnish Sharma, Vinay Kumar, Parul Sharma, Pankaj Kumar, Mohammad Irfan

**Affiliations:** 1 Department of Biotechnology, Dr. Yashwant Singh Parmar University of Horticulture and Forestry, Solan, Himachal Pradesh 173230, India; chanchalbhardwaj804@gmail.com (C.K.); mghsharma54@gmail.com (M.S.); vinaybanyal123@gmail.com (V.K.); rajnish.sharma@yahoo.co.in (R.S.); pksharmabiotech@gmail.com (P.K.); 2 Department of Physiology and Cell Biology, Department of Internal Medicine, The Ohio State University Wexner Medical Center, Columbus, OH 43210, USA; vinayktyagi07@gmail.com; 3 Plant Biology Section, School of Integrative Plant Sciences, Cornell University, Ithaca, NY 14853, USA

**Keywords:** post-harvest loss, genetic engineering, genome editing, CRISPR/Cas9, horticultural crops, fruits, vegetables, shelf-life, texture, post-harvest pathogen

## Abstract

Food security and crop production are challenged worldwide due to overpopulation, changing environmental conditions, crop establishment failure, and various kinds of post-harvest losses. The demand for high-quality foods with improved nutritional quality is also growing day by day. Therefore, production of high-quality produce and reducing post-harvest losses of produce, particularly of perishable fruits and vegetables, are vital. For many decades, attempts have been made to improve the post-harvest quality traits of horticultural crops. Recently, modern genetic tools such as genome editing emerged as a new approach to manage and overcome post-harvest effectively and efficiently. The different genome editing tools including ZFNs, TALENs, and CRISPR/Cas9 system effectively introduce mutations (In Dels) in many horticultural crops to address and resolve the issues associated with post-harvest storage quality. Henceforth, we provide a broad review of genome editing applications in horticulture crops to improve post-harvest stability traits such as shelf life, texture, and resistance to pathogens without compromising nutritional value. Moreover, major roadblocks, challenges, and their possible solutions for employing genome editing tools are also discussed.

## 1. Introduction

Horticultural crops comprise all the fruits, vegetables, and ornamentals, the majority of which are of utmost economic status due to their larger contribution toward total agricultural production. Fruits and vegetables are the reservoirs of valuable and vital nutrients such as minerals, vitamins, fibers, carbohydrates, proteins, and organic acids of the human diet, while ornamentals are worth of aesthetic nature. However, the serious concern with these crops is that they are quickly perishable and can respire and transpire even after harvest, resulting in excessive ripening-associated softening during post-harvest storage [1,2]. Consequently, the relevant post-harvest losses or waste occur in these horticultural crops with varied responses amongst crops, climatic zones, and handling countries. These post-harvest losses can be described as the loss of food in terms of its quality, nutrition, seed viability, and market value taking place in the food chain from harvesting to consumption. Globally, this loss happens to be approximately 1.3 billion tons on an annual basis, and the problem is even more challenging in developing countries. For instance, in India, annually approximately 30–40 percent of fruits and vegetables produce is wasted because of this [1,3,4]. Conventional methods such as low temperature or cold storage and chemical treatment remained the only means of minimizing these losses for many years by extending the crops shelf life. However, issues at the level of low-temperature storage are due to inadequate storage set-up and capacity, lesser accessibility of farmers to storage units, and poor carriage [1,2]. The chemical treatment also affects produce quality and results in associated health risks if used in random way. Although breeders have made numerous attempts to improve the post-harvest quality traits such as nutritional content, storage duration, color, flavor, texture, and size in order to achieve high market value, time and the labor-intensive nature of traditional breeding programs limit their use. Conversely, novel technologies including genetic and genome engineering hold the immense potential to cater the post-harvest losses and quality efficaciously.

**Figure 1:**
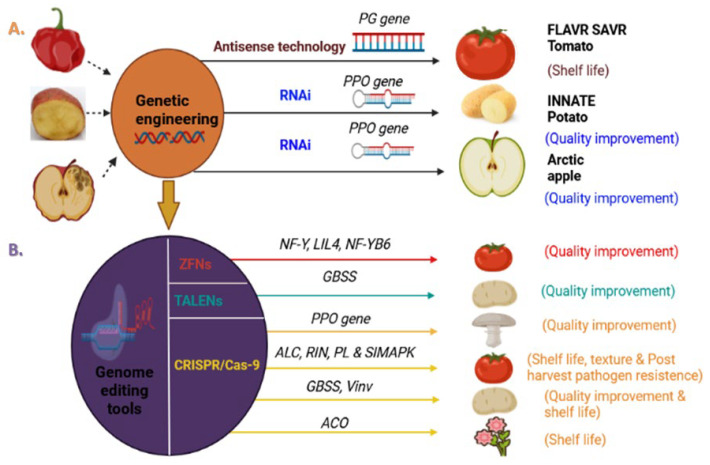
Biotechnological approaches improved post-harvest shelf life and quality of many horticultural crops: (**A**) anti-sense RNA (asRNA) and RNA interference (RNAi) technologies were used to enhance shelf life and quality in tomato, potato, and apple by targeting different genes, *PG* (polygalacturonase) and *PPO* (polyphenoloxidase), which showed various limitations such as off-target effect and concerned safety assessments. The arrow from (**A**) to (**B**) depicts the transition from biotechnological tools, i.e., genetic engineering to modern genome editing tools. (**B**)In contrast, advanced biotechnological approach, i.e., genome editing tools such as ZFNs, TALENs, and CRISPR/Cas-9 successfully modified the important post-harvest traits such as shelf life, texture, quality improvement, and post-harvest pathogen resistance.

Over the last few decades, genetic engineering techniques have been used tremendously to develop genetically modified (GM) crops by introducing genes of trait of interest to reduce post-harvest losses and improve the quality of a particular crop [5]. Utilizing its approaches such as anti-sense RNA (asRNA) and RNA interference (RNAi), post-harvest losses have been addressed in many crops [6] (Figure 1). Anti-sense RNA technology employs antisense sequences that are complementary to the target sense RNA strand and act as a regulatory molecule by binding to the target sense strand via base pairing and inhibiting gene expression. For example, in tomato and potato for increasing shelf life [7,8,9]; in flower crops such ascarnation and petunia for increasing senescence or vase life [10,11]; and in strawberry and tomato for preventing softening [12,13,14,15,16,17,18]. RNA interference (RNAi) technology, on the other hand, is based on the insertion of short sequences of double-stranded RNA (dsRNA), small interference RNA (siRNA), or hairpin RNA (hpRNA), which results in post-transcriptional gene silencing. This method has emerged as a promising strategy for reducing post-harvest losses such as in tomato and capsicum for increasing shelf life, preventing softening, and resistance against post-harvest pathogens [3,4,19,20,21,22,23,24]; in strawberry for improving shelf life and resistance against post-harvest pathogens [25,26]; in banana fruit crop for delayed ripening [27]; and in potato for increasing shelf life and improving appearance [28]. Some popular examples (Figure 1) of these approaches include flavrsavr tomato [7], Arctic apple [29], and innate potato [30]. Yet, this technology is still trapped in realizing its factual potential owing to the foreign nature of the gene of interest and uncertainties associated with health, environment, and overall public acceptance. In addition, the in-depth assessment of safety and the regulatory approach to applications of the technology deserves more attention [31,32,33]. To overcome these challenges, genomeediting technology has emerged as a breakthrough technology that has been effectively utilized to alter plant genomes without the introduction of foreign genes. The major advantage of this novel technology is the capability of editing an organism’s DNA through making precise modifications to DNA sequences in an efficient way [34,35].

Different genomeediting tools such as zinc-finger nucleases (ZFNs) [36], transcription activator-like effector nucleases (TALENs) [37], and Clustered Regularly Interspaced Short Palindrome Repeats (CRISPR)/CRISPR-associated protein 9 (Cas9) [38] have been widely used to improve the quality of various crops. These tools make use of endonucleases to introduce site-specific double-stranded DNA breaks (DSBs), and in turn, plant’s internal DNA repair mechanisms can mend these DSBs either via non-homologous end-joining (NHEJ) or homology-directed/dependent recombination (HDR). Repair via NHEJ results in alleles being knocked out via random insertions or deletions, while HDR leads to insertion of specific sequences specified by repair templates supplied in trans [39,40]. In ZFNs and TALENs, two domains, i.e., nuclease domain based on type II restriction endonuclease *Fok*I and DNA binding domain of ZF or TALE proteins are fused. Relying on their protein–DNA interaction, DSBs is created. On the other hand, CRISPR/Cas9 is adapted from a bacterial (*Streptococcus pyogenes*) defense mechanism against pervading bacteriophages or viruses where it acts by cleaving the foreign DNA in a sequence-dependent manner. The CRISPR/Cas9 system transitioned from its important role in bacterial immunity to a genome editing tool when its reprogramming capacity was exploited by altering a few base pairs (20) of single guide RNA (sgRNA). Following numerous studies, it evolved into a genome editing tool that depends on RNA-DNA binding. In this system, the non-specific Cas9 endonuclease and specific sgRNA/gRNA (single guide RNA) act synergistically. sgRNA/gRNA directs Cas9 which requires a *protospacer adjacent motif* (PAM) sequence for specific cleavage in target DNA, thereby causing the DSBs [40,41,42]. CRISPR/Cas9 is a more significant editing tool in comparison to ZFNs and TALENs, attributing to its characteristics such aspreciseness, cost-effectiveness, reprogramming ability, and applicability. Similar to genetic engineering, this technology utilizes genetically encoded delivery of CRISPR/Cas9 components into plant cells to make the precise alterations in the host genome. This can be achieved either by means of widely used biolistic gun or *Agrobacterium*-mediated methods. However, these methods possess certain limitations like gene silencing and positional effects due to the involvement of promoters, terminators, selectable marker genes, T-DNA, etc. [43]. Thus, the incorporation of foreign recombinant DNA fragments into plant genomes makes them eligible for GMO regulation. On the other hand, the DNA-free preassembled Cas9/gRNA ribonucleoprotein (RNP) complex cleaves the target sites immediately after delivery and rapidly degrades, with lower or negligible off-targeting rates possibly due to the short lifetime of the introduced CRISPR/Cas9 complex. When compared with abovementioned DNA-based delivery methods of the Cas9/gRNA complex, the continual synthesis of functional enzymes could be held responsible for off-targeting frequency [44]. That being said, many RNP delivery approaches have been developed to deliver CRISPR/Cas9 and its components in cases of plants and animals such as physical (microinjection, biolistic, polyethylene glycol (PEG), electroporation, microfluidics, filtroporation, nanotube, protoplast transformation, lipofection) and carrier-based (virus-like particles, lipid nanoparticles, lipopeptides, polymers, CPPs (Cell-Penetrating Peptides), nanogels, inorganic nanoparticles (gold nanoparticles, graphene oxide, calcium phosphate nanoparticles, etc.)). Most methods such asa biolistic gun and PEG-mediated protoplast transfection have found their applicability for RNP delivery in the case of plants as well, with only very few reports on lipofection, electroporation, lipid nanoparticles, etc. [45]. Recently, this technology has been acknowledged globally and awarded Nobel Prize in the year 2020. To make this technology more effective and user-friendly, various software and websites have been developed such as CRISPR-Plant, CRISPRdirect, GT-Scan, CrisprGE, Cas-OFFinder, CRISPy-Web, Prime Design, pegFinder, CRISPOR, Genome CRISPR, SSFinder, CHOPCHOP, CRISPR-P, RGEN BE-designer, RGEN Cas designer, etc. [46,47]. The key steps in CRISPR/Cas9 editing system entail: (a) software-aided exploration of the target sequence; (b) scheming the artificial gRNAs and respective components through computerized tools; (c) transfer of CRISPR based gRNA complex in a respective host using a competent delivery system; (d) analyzing the proficiency of CRISPR/Cas9 editing via assays, viz., T7 endonuclease I-based mismatch cleavage, sequencing based on TIDE (Tracking of Indels by Decomposition), cell-based Site-Seq, NGS (Next-Generation Sequencing), and FACS (fluorescence-activated cell sorting); and (e) assessment of edited plants based on phenotype. Apart from CRISPR/Cas9, additional systems such as CRISPR/Cpf1 or Cas12a were also re-purposed from bacterial species *Prevotella* and *Francisella* for genome editing [34,40,48].

Globally, the crops with modified genomes are categorized into three classes: Site-Directed Nucleases-1 (SDN-1), Site-Directed Nucleases-2 (SDN-2), and Site-Directed Nucleases-3 (SDN-3) based on their editing behavior (mutation type/donor DNA) and respective repair mechanisms. SDN-1 application utilizes endogenous NHEJ pathway to repair the DSBs and causes gene knockout or knockdown by random mutation in the form of insertions and deletions (In Dels). SDN-2 relies on HDR repair with desired sequence modification at the DSB target site by using homologous template DNA (short) leading to gene modification (gain of function). In contrast to SDN-1 and SDN-2, SDN-3 makes use of the HDR pathway by incorporating the new gene or DNA sequence leading to gene insertion [49,50,51].

**Figure 2:**
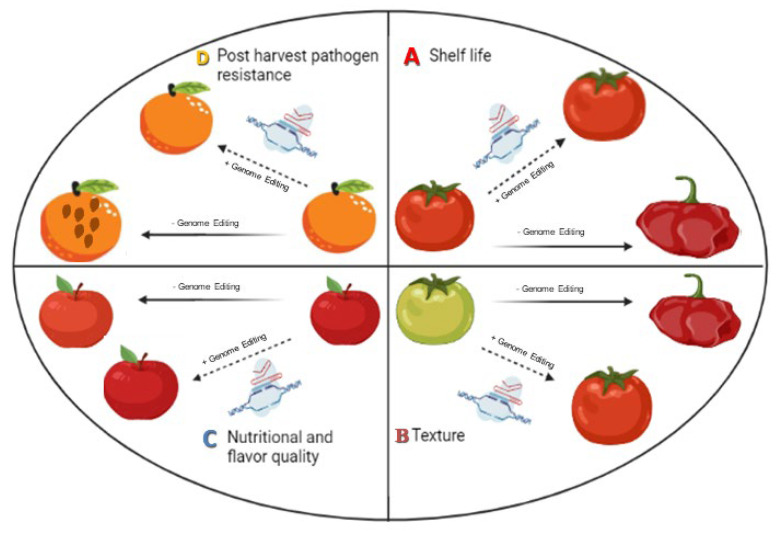
Genome editing tool could enhance various parameters of post-harvest shelf life and quality of horticultural crops in comparison to normal conditions: (**A**) It may extend shelf life and delay ripening without affecting post-harvest quality. (**B**) It may maintain the fruit texture without altering other characters such asfruit size and color. (**C**) Nutritional and flavor quality may be enhanced without losing post-harvest quality. (**D**) Post-harvest diseases could be overcome.

In recent years, genomeediting technology has become more widely used for crop improvement with the primary goals being to improve the nutritional and functional qualities of various crops not only in the field but also during the post-harvest storage, e.g., in tomato, potato, mushroom, apple, and petunia, etc. (Figure 1). In this review, we discuss up-to-date information on how genome editing technology was used to alter the biological processes that control the quality traits of fruits, vegetables, and ornamentals including shelf life, texture, resistance to pathogens, and nutritional and flavor enhancement, particularly during post-harvest storage (Figure 2).

## 2. Genome Editing for Enhancing Post-Harvest Quality Attributes

### 2.1. Enhancement of Shelf Life

Longer shelf life is one of the most key traits for fleshy fruits, vegetables, and ornamentals, and it has a greater impact on market potential. Maintaining and prolonging the shelf life is a major challenge in breeding and genome engineering programs. Cold storage has been used to extend the shelf-life of these crops for several years, but this strategy is prohibitively expensive for smallholder growers. Therefore, there is an urgent need to develop strategies to generate crops with a longer shelf-life. The plant hormone ethylene, also known as the ripening hormone, plays a vital role in the ripening process of fruits and vegetables; therefore, its production needed to be controlled to maximize the shelf-life [52,53,54,55]. Under natural conditions, this ripening process ultimately leads to senescence. Therefore, to lessen the effects of post-harvest losses, some management strategies need to be devised to delay this ripening process during transportation and storage. Meanwhile, this path of horticulture produce from ripening to senescence stage is responsible for decreased quality, which ultimately leads to its rejection at the consumer end causing the related post-harvest losses. Hence, it becomes imperative to regulate the expression of shelf-life-related genes to maintain the taste, aroma, and quality features [52,53,56]. Recently, advanced genome editing technology tools have been potentially utilized to enhance the quality as well as post-harvest traits of horticultural crops (Table 1). Tomato, being a model climacteric crop, is the most scientifically investigated for genome editing studies as it was the first crop that had been manipulated through genetic engineering because of the availability of molecular-level information related to shelf-life processes.

The *RIPENING INHIBITOR (RIN), COLORLESS NONRIPENING (CNR)*, and *NONRIPENING (NOR), Alcobaca (ALC)* genes encode transcription factors regulating the fruit ripening in various climacteric and non-climacteric species [52,57,80]. The *RIN* gene was disrupted by using CRISPR/Cas9 genome editing in a tomato cultivar Ailsa Craig, resulting in slower ripening accompanied by less lycopene pigment production with enhanced shelf life [58,81]. Furthermore, CRISPR/Cas9 was also used to target the long non-coding RNA-1459 (lncRNA1459) in tomatoes, which resulted in mutants with reduced ethylene production, lycopene assimilation, and significantly contributed to delayed ripening [82]. The introduction of *ALC* gene template (substitution of thymine by adenine) using sgRNA-CRISPR/Cas9 vector followed by DSBs repair by HDR pathway resulted in the longer shelf life of gene-edited homozygous recessive tomatoes showing significant storage rates [59]. Mutants showing incomplete non-ripening behavior by editing the *NOR* gene were developed [22,60]. Further, these mutants were evaluated for fruit ripening response, and it was inferred that few ripening-related transcription factors and genes were responsible for fruit ripening, i.e., *SlACS2, Sl Ggpps2,* and *SlPL* [83]. From various studies, it can be concluded that *CNR* is not the dominant fruit ripening regulator and needs more evaluation in this regard. In addition to this, *CNR* mutants displayed only 2–3 days delayed ripening response [60].

Another important food crop potato is studied for post-harvest management to increase its shelf life. In the case of potatoes, two types of browning decrease the value of its processed products: one is non-enzymatic, and the other is enzymatic. Non-enzymatic browning occurs under cold storage conditions where the stored sugars are converted into their reduced form. This phenomenon is known as cold-induced sweetening (CIS). Upon exposing the potato tubers to high temperatures, they undergo browning due to reaction between reduced sugars and the free form of amino acids leading to generation of acrylamide, which is a potent carcinogen. Enzymatic browning is caused by polyphenol oxidase (*PPO*) enzyme-based phenols to quinones conversion. The bioengineering tools such as genome editing in potatoes started with the application of TALEN-induced editing that aimed to knockout the genes (vacuolar invertase, *Vinv*) involved in CIS process in the case of Ranger Russet potatoes. Only five knockouts of four related *Vinv* alleles exhibited the relevant responses, viz., no reducing sugars and decreased acrylamide levels with light brown tubers. It was made commercially available by Calyxt Inc., formerly known as Cellectis Plant Sciences [35,61]. Additionally, TALEN-based knocking out of browning genes (*PPO*) using *Agrobacterium* and PEG-mediated transformation methods in potatoes was achieved by the US-based companies, namely, Calyxt Inc. and Simplot Plant Sciences, with reduced tubers browning [84], while the enzymatic browning was addressed in the Desiree potato cultivar using a CRISPR/Cas9-based RNPs system to edit the *StPPO2* (polyphenol oxidase) gene to produce potato regenerants with alleviated PPO activity and enzymatic browning (69% & 73%), respectively [62]. Likewise, in mushroom (*Agaricus bisporus*), the enzymatic browning was decreased to 30% through CRISPR/Cas9-mediated knocking out of *PPO* gene that significantly resulted in improved shelf life, thereby enhancing its overall quality [63]. It gained immense popularity as it escaped the regulation process of USDA and became the first genome-edited crop to attain this status [63]. These studies formed a firm base toward the application of these cutting-edge genome editing tools (TALENS and CRISPR/Cas9) to genetically ameliorate the horticultural crops concerning their post-harvest quality attributes.

### 2.2. Fruit Texture Quality Improvement

As far as post-harvest stability is concerned, texture remained a vital attribute in the case of horticulture crops. Texture-related number of enzymes such as polygalacturonase (*PG*), pectin methylesterase (*PME*), endo-b-(1,4)-glucanase (*EGs*), β-galactosidase (*β-gal*), and expansin (*EXPs*) and N-glycoprotein-modifying enzymes, e.g., α-mannosidase (*α-Man*) and β-D-N-acetylhexosaminidase (*β-Hex*) are responsible for firmness and softening processes in these crops [3,4,23,24,85]. Various research reported the suppression of relevant gene expression in strawberries and tomatoes [13,85]. In tomato, *PG* gene suppression had no obvious effect on fruit softening [12], but this gene also influences the firmness of strawberries with higher Brix to some extent [25]. However, another gene, i.e., pectate lyase (*PL*) gene, which is a cell wall-related protein has been silenced (asRNA approach) effectively to enhance fruit firmness without changing its physical (size and color) and biochemical (total soluble solids, metabolites, etc.) parameters, ultimately influencing the sensory characteristics in strawberry [15] and tomato [65], respectively. In this process, utilizing CRISPR/Cas9 editing of *PL* gene resulted in the mutants exhibiting a beneficial effect on fruit firmness while maintaining the fruit color, aroma, and flavor in tomato [65] (Table 1).

### 2.3. Improving Post-Harvest Pathogen Resistance

Post-harvest infections are the major concern to fruits, vegetables, and ornamentals from ripening and harvesting to their transportation from field to farm, processing units, and storage chambers. Upon the storage of horticultural crops, abiotic factors, viz., temperature, relative humidity, and oxygen balance, greatly contribute toward their receptivity to pathological attacks. The pathogens mainly responsible for post-harvest losses include fungi, bacteria, yeast, and molds [86]. During post-harvest management of horticultural crops, the various pathogens, bacterial and fungal rots (Table 2) [87,88] are the most devastating, as they cause serious harm to perishables and canned products. Bacterial and fungal rot deteriorates the majority of fruits and vegetables [86]. The major causal bacterial soft rots agents are various species of *Erwinia, Pseudomonas, Bacillus, Lactobacillus,* and *Xanthomonas*. However, fungus infections, which cause rot in fruits and vegetables, are more common than bacteria during various post-harvest processes. Considerable post-harvest losses are caused by *Alternaria, Aspergillus, Botrytis, Colletotrichum, Diplodia, Dothiorella, Fusarium, Monilinia, Mucor, Penicillium, Phomopsis, Phytophthora, Pythium, Rhizoctonia, Rhizopus, Sclerotium,* etc. Apart from high temperatures and relative humidity conditions responsible for post-harvest pathogens development, the acid content of fruits and vegetables also has an important impact on pathogen attacks. For example, those with more acid content (low pH) are generally attacked by fungi, whereas those bearing a pH of more than 4.5 get attacked by bacterial pathogens [89,90]. For the management of post-harvest diseases, procedures for disease management (pesticides such as bactericides, nematicides, insecticide), cropharvest (cushioning), transport (ventilated and temperature controlled), storing (cold temperature, spacious chambers), pre- and post-harvest treatments (chemicals such as sulfur dioxide, benzoic acid, ascorbic acid, calcium chloride, etc., and UV-C treatment) are routinely utilized. More appropriate measures in context to these strategies such as compatible microbial formulations, excellent cushioning material, automated cooling units/chambers, UV-C treatment, etc., can limit the extent of post-harvest loss in horticultural crops [91,92]. So far, genetic engineering has been widely used to improve disease (insect, fungal, bacterial, viral, insect) resistance in horticultural crops by incorporating various genes such as *Cry* genes, protease inhibitors, trypsin inhibitors, PR proteins, defensin, thionins, chitinase, glucanase, osmotin, cystatin, cp, etc. [93,94]. New gene-editing methods make it easier to produce new crop types with improved biotic stress response [95].

In citrus, mostly the economic damages are associated with the bacterium *Xanthomonas citri* subsp. *Citri*, causing citrus canker with the occurrence of severe symptoms in stem, leaf, and fruit. This disease is of concern because of its appearance at pre- and post-harvest levels. To alleviate the losses on account of this pathogen, the relevant transcription factor, i.e., *LATERAL ORGAN BOUNDARIES 1 (CsLOB1)*, responsible for disease manifestation was knocked out through the CRISPR/Cas9 approach [66]. In Duncan grapefruit (*Citrus paradisi*), by targeting the susceptibility gene, i.e., *CsLOB1* CRISPR/Cas based mutants (*DLOB9* and *DLOB10*) were produced with improved resistance toward *Xanthomonas citri* [67]. Further, Peng et al. [103] conducted CRISPR/Cas9 based susceptibility (S) gene editing in *Citrus sinensis* Osbeck to develop canker-resistant mutant plants. Likewise, tomato is another crop that is prone to post-harvest pathogen attack in the form of *Botrytis cinerea* (gray mold), causing major economic losses. CRISPR/Cas9-mediated targeting of *SIMAPK3* gene susceptible for gray mold was attained to produce tomato mutants with increased resistance by enhanced expression of secondary metabolites of defense pathways and reactive oxygen species (ROS) accumulation regulation [68]. To overcome the post-harvest losses in grapes, CRISPR/Cas9 strategy-based knocking out of the *VvWRKY52* transcription factors linked to biotic stress was achieved by developing mutants possessing resistance to *Botrytis cinerea* without any significant change in mutant plant phenotype [69]. The causal organism of Anthracnose is *Colletotrichum truncatum*, which causes major pre- and post-harvest losses in *Capsicum annuum*. Therefore, susceptibility (S) gene ethylene-responsive factor (*CaERF28*) was modified using Cas9/sgRNA cascade to produce mutant lines with elevated resistance against anthracnose, revealing the proper expression of defense-related genes [70]. GM applications can prevent Hualongbing (citrus greening disease caused by *Liberibacter asiaticus* by the expression of antibacterial compounds and defensins in crops such as orange trees [104,105,106].

### 2.4. Nutritional and Flavor Quality Enhancement

Horticultural crops possess abundant nutrients, namely, vitamins, minerals, dietary fibers, antioxidants, etc. Their flavor and quality are highly influenced by many factors including genetic composition, field conditions, mode of harvesting, and post-harvest management. These perishable crops lose their peculiar edible features (taste, aroma, nutritive contents, etc.), leading to an inedible state (offflavors) depending upon their extended conditions after harvesting until consumption. Thus, it is important to produce the best-tasting genotypes using improved and advanced approaches to maintain optimal flavor and nutritional quality of horticultural crops beginning with harvesting until consumption. CRISPR/Cas9 genome editing in horticultural crops is an evolving field, hence, with only a few findings in context to nutritional and flavor quality to date, as represented in Table 1.

Using ZFN technology, the function of nuclear transcription factor Y (*NF-Y*) transcription factor (TF) gene *LEAFY-COTYLEDON1-LIKE4 (L1L4, NF-YB6)* was disrupted in tomatoes to produce *L1L4* mutants. In comparison to wild types, few mutants exhibited variation in metabolic contents such as oxalic acid, citric acid, fructose, β-carotene, total phenols, and antioxidants. It was inferred that in tomato fruit and seeds, *L1L4* TF is a key regulator of biosynthetic pathways of seed storage proteins and fatty acids [71]. This research is performedto identify targets for use without practical applications at hand.

TALENs-based targeting of starch branching enzyme (*SBE1*) and acid invertase (*INV2*) in potato cultivars Russet Burbank and Shepody led to respective mutations, thereby impacting the degree of starch branching and cold-induced sweetening (CIS) [72].

CRISPR/Cas9 system edited the L-idonate dehydrogenase (*IdnDH*) gene responsible for constant tartaric acid (TA) accumulation revealing no off-target mutations, suggesting the effective applicability of this system in grape [73]. Subsequently, this approach modified the *IdnDH* gene in grape and apple [74]. In another study of *Solanum pimpinellifolium* (currant tomatoes), CRISPR–Cas9 was used for editing the upstream open reading frames (uORFs) of genes associated with morphology, flower and fruit production, and ascorbic acid synthesis. The content of vitamin C in edited tomatoes was shown to be higher, and uORF was discovered to be another suitable target for genome editing [82]. By editing starch synthesis granule-bound starch synthase (*GBSS*) gene using CRISPR/Cas9 in potato, only four mutants displayed knockout of alleles of *GBSS* gene related to amylopectin production [75]. Likewise, the same gene was edited using CRISPR/Cas9 with mutants showing low levels of amylose starch [76]. Furthermore, mutagenesis of starch-branching enzymes (*SBE1* and *SBE2*) by CRISPR/Cas9 generated new, potentially valuable starch properties in potatoes. During granule growth, amylopectin branching was reduced with the reduction in both *SBE1* and *SBE2* expression, whereas starch granule initiation was affected by *SBE2* [77]. The CRISPR/Cas9-based editing via cytidine base editor (CBE) resulted in the loss of function of N- terminal motif of *GBSSI* (*KTGGL*) gene, producing mutants with different amino acid sequences and resultant reduced biosynthesis of amylose [78]. γ-aminobutyric acid (GABA) possesses neurotransmitting activity, which helps in relaxing and lowering the blood pressure. Sanatech Seed’s company developed tomato variety “Sicilian Rouge” with high GABA content (4–5-fold) using the CRISPR/cas9 approach by editing the pathway of GABA synthesis. This involved GABA shunt, i.e., the disruption of the calmodulin-binding domain (*CaMBD*) genes to increase the activation of glutamic acid decarboxylase enzyme, which catalyzes the decarboxylation reaction for the conversion of glutamate to GABA [79].

## 3. Obstacles, Challenges, and Solutions

To properly understand the distinctiveness of the CRISPR Cas9-based genome editing tool, some major issues concerning its methodological aspects and application in horticultural crops must be addressed. Some of the challenges associated with genome editing include the following: (a) The complete sequence information as a foremost requirement to initiate genome editing work. However, particularly in many horticultural crops, it is unavailable, which becomes challenging and limits its broader applicability. (b) The efficient and reproducible in vitro regeneration protocol and gene transfer methodology including particle bombardment, PEG-mediated transformation, *Agrobacterium*-mediated, etc., have not been devised in many crops due to their recalcitrance nature toward plant tissue culture methods. Besides this, the lengthy and tedious procedures of desirable transformed/mutated plant selection and regeneration are another factors of consideration.(c) Some of the horticultural crops also possess complex genomic structures leading to their inaccessibility for genomic studies.(d) The genes pertaining to post-harvest quality traits remains unstudied to a greater extent because of their quantitative nature.(e) The regulatory uncertainty in many countries due to ambiguities of the current bio-safety frameworks restricts the applicability of this technology in context to genetically edited crops (SDN-1, SDN-2, and SDN-3) [107]. In addition, the proponents of the technology are concerned by “overregulation” according to the current GMO-laws, and not by a lack of a specific regulatory framework.

Therefore, a greater number of crops need to be sequenced to harness this technology. Suitable transformation and delivery protocols have to be developed in the case of recalcitrant crops for the generation of desired plants generation. Moreover, using DNA-free genome editing technique based on ribonucleoprotein (RNP) complex (Cas9+ sgRNA) provided success in generating mutants in the case of grapes and apple [108] and potato [109] by PEG-mediated protoplast transfection or biolistic gun.

The regulation for genetically engineered crops has already been formulated and adopted by many countries. Most countries apply the existing regulatory frameworks for GMOs based on a case-by-case determination of the regulatory status of genome-edited organisms [107]. However, as the regulatory triggers for the existing bio-safety laws differ between different legislations and different options to address/include genome-edited organisms are pursued by different countries, the global regulatory landscape for genome-edited organisms is far more heterogeneous than for classical GMOs (transgenic organisms). Even international bodies such as the OECD (The Organization for Economic Co-operation and Development), which are working toward harmonization of regulatory oversight of biotechnology, conceded that fact. It is hard to see how better coordination among scientists and a better understanding of these technologies would overcome this situation. Overall, the adoption of appropriate regulations needs to be accelerated effectively to reap the real capability of this genome editing technology in the crop improvement program to cater the high-quality, nutrient-rich food requirements with accessibility to the burgeoning population across the world.

## 4. Conclusions

The ever-evolving advancements in science and technology contribute toward improving the traditional methods to keep pace with the developments occurring for the benefit of mankind. The global changing scenario necessitates quick and feasible solutions to meet the growing population’s food needs with improved nutritional value. This could be achieved in horticultural crops by utilizing the latest and promising technologies includinggenome editing, as documented in various studies in the case of numerous crops. However, to overcome some of the associated concerns, it needs a transparent, uniform regulatory system (same approach for all genome-edited organisms and GMOs) that can substantiate its broader applicability with safety and public acceptability.

## Figures and Tables

**Table 1:** Application of genome editing techniques in horticultural crops to improve their post-harvest quality and life.

S.N.	Crop Species	Gene Editing Tool	Transformation Method	Target Gene	Function of Target Gene	Outcome	Reference
**Shelf life**							
1.	Tomato	CRISPR/Cas9	*Agrobacterium tumefaciens*-mediated transformation	*ALC*	Inhibit ethylene synthesis (SN1 is an insertion of an actual inhibitor gene *ALC*)	Mutants with longer shelf life as compared to wild type	[57]
2.	Tomato	CRISPR/Cas9 (SDN1)	*Agrobacterium tumefaciens*-mediated transformation	*RIN*	Inhibit ethylene synthesis and specific biochemical processes related to fruit ripening	Mutant lines exhibited lower ethylene contents and delayed fruit ripening	[58]
3.	Tomato	CRISPR/Cas9 (SDN2)	*Agrobacterium tumefaciens*-mediated transformation	*ALC*	Inhibit ethylene synthesis (SN2 seems to be a knockout mutant of the RIN gene)	Mutants with longer shelf life as compared to wild type	[59]
4.	Tomato	CRISPR/Cas9 (SDN1)	Not mentioned	*SBP*-*CNR&NAC*-*NOR*	Transcription factor of ripening genes	Mutants displayed partial non-ripening phenotypes	[60]
5.	Potato	TALEN (SDN1)	Protoplast transfection using PEG mediated transformation system	*Vinv*	Hydrolyzes the sucrose produced from starch breakdown into one molecule of glucose and one of fructose	Mutant lines with improved cold storage and processing traits	[61]
6.	Potato	CRISPR/Cas9 (SDN1)	Protoplast transfection with RNPs using PEG mediated transformation system	*StPPO2*	Catalyzes the oxidation of phenolic compounds into compounds into quinones (highly reactive form)	Mutant lines exhibited reduction in enzymatic browning and *PPO* gene.	[62]
7.	White button mushroom	CRISPR/Cas9 (SDN1)	Protoplast transfection using PEG mediated transformation system	*StPPO2*	Catalyzes the oxidation of phenolic compounds into quinones (highly reactive form)	Mutants lines showed 30% reduction in enzymatic browning with improved appearance and shelf life	[63]
8.	Petunia	CRISPR/Cas9 (SDN1)	*Agrobacterium tumefaciens*-mediated transformation	*PhACO*	Catalyzes aminocyclopropane-1-carboxylic acid to ethylene in ethylene biosynthesis pathway	Mutant lines exhibited significant reduction in ethylene production and enhanced flower longevity as compared to wild-type	[64]
**Texture**							
9.	Tomato	CRISPR/Cas9 (SDN1)	Not mentioned	*PL*	Involved in plant cell wall degradation	Higher fruit firmness efficiency were found in mutants plants	[65]
**Post-harvest pathogen resistance**							
10.	Citrus	CRISPR/Cas9 (SDN1)	*Agrobacterium tumefaciens*-mediated transformation	*CsLOB1*	Disease susceptibility gene for citrus bacterial canker	Mutant lines showed lower host pustule development with improved fungal resistance against *Xanthomonas citri* subsp.*citri*.	[66]
11.	Citrus	CRISPR/Cas9 (SDN1)	*Agrobacterium tumefaciens*-mediated transformation	*CsLOB1*	Disease susceptibility gene for citrus bacterial canker	Improved fungal resistance against citrus bacterial canker in mutant plants	[67]
12.	Tomato	CRISPR/Cas9 (SDN1)	Not mentioned	*SlMAPK3*	*MAPKs* genes play an important role in defense responses to biotic and abiotic stresses	Mutants lines were prepared by knocking out *SIMAPK3* gene that showed resistance to *Botrytis cinerea*	[68]
13.	Grape	CRISPR/Cas9 (SDN1)	*Agrobacterium tumefaciens*-mediated transformation	*VvWRKY52*	Important in plant biotic stresses responses	Mutants lines with knocked out *VvWRKY52* gene showed higher resistance to *Botrytis cinerea*	[69]
14.	Chili pepper	CRISPR/Cas9 (SDN1)	*Agrobacterium tumefaciens*-mediated transformation	*CaERF28*	Susceptibility gene for anthracnose disease	Mutant lines showed higher resistance toward anthracnose	[70]
**Nutritional and flavor quality**							
15.	Tomato	ZFNs (SDN1)	Not mentioned	NF-Y, *L1L4*, *NF*-*YB6*	Responsible for biosynthesis for seed storage proteins and fatty acids	Mutants showed varied metabolite profiles and high amounts of OA as compared to wild type	[71]
16.	Potato	TALEN (SDN1)	*Agrobacterium tumefaciens*-mediated transformation	*SBE1* and *INV2*	*SBE1* enzymes are responsible forformation of amylopectin. *INV2* catalyze the irreversible hydrolysis of sucrose into glucose and fructose	Improved amylopectin content and cold sweetening	[72]
17.	Grape	CRISPR/Cas9 (SDN1)	*Agrobacterium tumefaciens*-mediated transformation	*IdnDH*	Important enzyme in tartaric acid (TA) biosynthetic pathway	Significant accumulation of tartaric acid (TA) in mutants lines	[73]
18.	Apple	CRISPR/Cas9	*Agrobacterium tumefaciens*-mediated transformation and PEG transformation system	*IdnDH*	Important enzyme in TA biosynthetic pathway	Stable accumulation of TA in mutant plants	[74]
19.	Potato	CRISPR/Cas9 (SDN1)	Protoplast transfection using PEG mediated transformation system	*StGBSS*	Responsible for amylase synthesis	Mutant lines showed higher amylopectin content than wild type	[75]
20.	Potato	CRISPR/Cas9 (SDN1)	*Agrobacterium tumefaciens*-mediated transformation	*StGBSS*	Responsible for the synthesis of amylase in starch biosynthetic pathway	Improved amylopectin content in potato plants	[76]
21.	Potato	CRISPR/Cas9 (SDN)	*Agrobacteriumtumefaciens*-mediated transformation and PEG transformation system	*SBE1*, *SBE2*	Starch branching enzymes which introduce α -1,6 -linkages into starch	Mutant lines showed reduced amylopectin content during granule growth	[77]
22.	Potato	CRISPR/Cas9	*Agrobacterium tumefaciens*-mediated transformation	*StGBSS*	Responsible for the synthesis of amylase in starch biosynthetic pathway	Mutant plants showed higher amylopectin content by using a CBE	[78]
23.	Tomato	CRISPR/Cas9 (SDN1)	*Not mentioned*	*CaMBD*		Improved GABA content (4–5 times)	[79]

CRISPR/Cas9: clustered regularly interspaced short palindromic repeats/CRISPR associated 9; PEG: polyethylene glycol; *ALC*: alcobaca gene; *RIN:* ripening inhibitorgene; *StPPO2*: solanum tuberosumpolyphenol oxidase 2 gene; PPO: polyphenol oxidase TALEN: transcription activator-like effector nucleases; *Vinv*: vacuolar invertase genes; *PhACO*: *petunia hybrida*1-aminocyclopropane-1-carboxylateoxidase genes; *PL:* pectate lyase gene; *CsLOB1*: *citrus* spp.transcription factor *LATERAL ORGAN BOUNDARIES 1; VvWRKY52:vitis vinifera* WRKY transcription factor; *SlMAPK3*: *solanum lycopersicum* mitogen-activated protein kinases; *CaERF28: capsicum annuum* ethylene-responsive factor gene; *IdnDH:* L-idonate dehydrogenase gene; TA: tartaric acid; *StGBSS*: solanum tuberosum granule-bound starch synthase gene; *SBE1:* starch branching enzyme 1; *INV2*: acid invertase gene; ZFNs: zinc-finger nucleases; *NF-Y*: nuclear transcription factor Y; *L1L4*, *NF*-*YB6:* transcription factor gene *LEAFY-COTYLEDON1-LIKE4;* OA: oxalic acid CBE: cytidine base editor; SBE2: starch branching enzyme 2.

**Table 2:** Major pathogens causing the post-harvest losses in important fruit and vegetable crops.

Crop	Disease	Causal Pathogen	Reference
**Fruit crops**			
Pome Fruit	Blue mold Gray mold Bitter rot Alternaria rot Mucor rot	*Pencillium* spp. *Botrytis cinerea* *Colletotrtchum gloeosporioides* *Alternaria* spp. *Mucor piriformis*	[87]
Stone Fruit	Brown rot Rhizopus rot Graymold Blue mold Alternaria rot	*Monilia* spp. *Rhizopus* spp. *(mostly* *R. stolonde)* *Botrytis cinerea* *Penicillium* spp. *Alternaria alternate*	[96]
Berries	Graymold Rhizopus rot Cladosporium rot Blue mold	*Botrytis cinerea* *Rhizopus* spp. *Cladosporium* spp. *Pencillium* spp.	[96]
Mango	Anthracnose Stem end rot Rhizopus rot Black mold Alternaria rot Graymold Blue mold Mucor rot	*Colletotrichum gloeosporioides, C. Acutatum* *Dothiorella* spp. *Phomopsis mangiferae* *Rhizopus stolonifer* *Aspergillus niger* *Alternaria alternate* *Botrytis cinerea* *Penicillium expansum* *Mucor circinelloides*	[96]
Papaya	Anthracnose Black rot Phomopsis rot Rhizopus rot Phytophthora fruit rot	*Colletotrichum* spp. *Phomacaricae-papayae* *Phomopsis caricae-papayae* *Rhizopus stolonifer* *Phytophthora palmivora*	[96]
Grapes	Blue mold Graymold Rhizopus rot	*Pencillium* spp. *Botrytis cinerea* *Rhizopus* spp.	[97]
Citrus Fruit	Blue mold Green mold Black center rot Stem end rot Brown rot	*Penicillium italicum* *Penicillium digitatum* *Alternartacitri* *Phomopsis citri* *Phytophthora citrophthora* and/or *P. Parasitica*	[98]
Avocado	Anthracnose Stem end rot	*Colletotrichum gloeosporoides, C. Acutatum* *Dothiorella*spp., *Lasiodiplodiatheobromae*	[99]
Banana	Anthracnose Crown rot Black end Ceratocystis fruit rot	*Colletotrichummusae* Various fungi including *Fusarium* spp., *Vertcillium* spp., *Acremonium sp*. and *Colletotrichum musae* Various fungi including *Colletotrichum musae, Fusarium* spp., *Nigrospora sphaerica* *Ceratocystis paradoxa*	[100]
**Vegetable crops**			
Carrot	Bacterial soft rot Rhizopus rot Watery soft rot Graymold Sclerotium rot	Various *Erwinia* spp. and *Pseudomonas* spp. *Rhizopus* spp. *Sclerotinia* spp. *Botrytis cinerea* *Sclerotium rolfsii*	[88]
Cucurbits	Bacterial soft rots Graymold Fusarium rot Alternaria rot Charcoal rot Cottony leak Rhizopus rot	Various *Erwinia* spp., *Bacillus polymgyxa*, *Pseudomonas syringae*, *Xanthomonas campestris* *Botrytis cinerea* *Fusarium* spp. *Alternaria* spp. *Macrophomina phaseolina* *Pythium* spp. *Rhizopus* spp.	[96]
Tomato, Eggplant, and Capsicum	Bacterial soft rots Graymold Fusarium rot Alternaria rot Cladosporium rot Rhizopus rot Watery soft rot Cottony leak Sclerotium rot	Various *Erwinia* spp., *Bacillus polymyxa*, *Pseudomonas* spp., and *Xanthomonas campestris* *Botrytis cinerea* *Fusarium* spp. *Alternaria* spp. *Cladosporium* spp. *Rhizopus* spp. *Sclerotinia* spp. *Pythium* spp. *Sclerotium rolfsii*	[96]
Brassicas, Leafy Vegetables	Bacterial soft rots Graymold Alternaria rot Watery soft rot Phytophthora rot	Various *Erwinia* spp., *Bacillus polymyxa*, *Pseudomonas* spp., and *Xanthomonas campestris* *Botrytis cinerea* *Alternaria* spp. *Sclerotinia*spp. *Phytophthora porri*	[96]
Onion	Bacterial soft rots Black mold rot Fusarium basal rot Smudge	Various *Erwinia* spp., *Lactobacillus* spp., and *Pseudomonas* spp. *Aspergillus niger* *Fusarium oxysporum* f. sp. *cepae* *Colletotrichum circinans*	[101]
Potato	Bacterial soft rot Dry rot Silver scurf	*Erwinia* spp. *Fusarium* spp. *Helminthosporium solani*	[102]

## Data Availability

Not applicable.
